# Comparison of Brain Gene Expression Profiles Associated with Auto-Grooming Behavior between *Apis cerana* and *Apis mellifera* Infested by *Varroa destructor*

**DOI:** 10.3390/genes15060763

**Published:** 2024-06-11

**Authors:** Jiali Liao, Kunlin Wan, Yang Lü, Wenyao Ouyang, Jingnan Huang, Liyuan Zheng, Liuchang Miao, Songkun Su, Zhiguo Li

**Affiliations:** 1 College of Bee Science and Biomedicine, Fujian Agriculture and Forestry University, Fuzhou 350002, China; 18960729081@163.com (J.L.); 18850731841@163.com (K.W.); 6220623004@fafu.edu.cn (Y.L.); oyooooung@163.com (W.O.); zly1328456253@163.com (L.Z.); miaolc0506@163.com (L.M.); 2 Mudanjiang Branch of Heilongjiang Academy of Agricultural Sciences, Mudanjiang 157000, China; 3 School of Life Sciences, Tsinghua University, Beijing 100084, China; hjn23@mails.tsinghua.edu.cn; 4 Academy of Bee Science, Fujian Agriculture and Forestry University, Fuzhou 350002, China

**Keywords:** *Apis cerana*, *Apis mellifera*, grooming behavior, *Varroa destructor*, RNA sequencing

## Abstract

The grooming behavior of honeybees serves as a crucial auto-protective mechanism against *Varroa* mite infestations. Compared to *Apis mellifera*, *Apis cerana* demonstrates more effective grooming behavior in removing *Varroa* mites from the bodies of infested bees. However, the underlying mechanisms regulating grooming behavior remain elusive. In this study, we evaluated the efficacy of the auto-grooming behavior between *A. cerana* and *A. mellifera* and employed RNA-sequencing technology to identify differentially expressed genes (DEGs) in bee brains with varying degrees of grooming behavior intensity. We observed that *A. cerana* exhibited a higher frequency of mite removal between day 5 and day 15 compared to *A. mellifera*, with day-9 bees showing the highest frequency of mite removal in *A. cerana*. RNA-sequencing results revealed the differential expression of the *HTR2A* and *SLC17A8* genes in *A. cerana* and the *CCKAR* and *TpnC47D* genes in *A. mellifera*. Subsequent homology analysis identified the *HTR2A* gene and *SLC17A8* gene of *A. cerana* as homologous to the *HTR2A* gene and *SLC17A7* gene of *A. mellifera*. These DEGs are annotated in the neuroactive ligand–receptor interaction pathway, the glutamatergic synaptic pathway, and the calcium signaling pathway. Moreover, *CCKAR*, *TpnC47D*, *HTR2A*, and *SLC17A7* may be closely related to the auto-grooming behavior of *A. mellifera*, conferring resistance against *Varroa* infestation. Our results further explain the relationship between honeybee grooming behavior and brain function at the molecular level and provide a reference basis for further studies of the mechanism of honeybee grooming behavior.

## 1. Introduction

*Varroa destructor* is a major ectoparasite and a prominent vector of viral pathogens and is considered to be the greatest threat to honeybee [1,2]. *Varroa* mites initially migrated from their native host (*Apis cerana*) to *Apis mellifera* and later spread to nearly all parts of the world [3]. For the control of *Varroa* mites, the most widely used products are synthetic acaricides, and the choice of acaricide varies by country and region [4,5]. These mite-killing drugs can also have hazardous side effects on honeybee colonies, such as leaving toxic residues in bee products and promoting acaricide resistance in mites [6].

Breeding *Varroa*-resistant honeybees has been a focal point for researchers and breeders worldwide [7,8]. *A. cerana* exhibit lower susceptibility to mites due to their long coexistence. The development of *Varroa* resistance in *A. cerana* occurs naturally through the process of natural selection, without human intervention [9]. In certain regions, like Mexico, Brazil, and Africa, beekeepers do not take any measures but resist *Varroa* mites infestations through natural selection. After the initial loss, honeybees (*A. mellifera*) gradually adapted to mites over time and became resistant through natural selection [10]. The anti-mite species, Africanized bees (hybrids of *A. m. scutellata*), appeared in South and Central America [11,12]. Grooming behavior plays a crucial role in the resistance mechanisms that have evolved within honeybee populations. Auto-grooming and allogrooming are essential behaviors among bees. Auto-grooming involves biting and licking with mouthparts, as well as foot-cleaning various parts of the body. However, if a single worker bee cannot eliminate a mite through auto-grooming alone, it will engage in a grooming dance to enlist the help of nearby worker bees. These surrounding bees inspect the parasitized worker’s body with their antennae, searching for the mite. Once located, they use their upper jaw to pick up and kill the mite [13,14,15].

Guzman-Novoa et al. described the both ‘light grooming’ and ‘intense grooming’ behaviors. Their results showed that bees from the resistant genotypes performed significantly more instances of intense grooming, and a significantly higher number of mites were dislodged from the bees’ bodies through intense grooming than through light grooming in all genotypes, suggesting that grooming intensity is an essential factor in resistance to *Varroa* mite [15]. The neural gene *AmNrx1* (*neurexin-1*), identified in a quantitative trait locus, exhibits significantly higher expression in honeybees that display intense grooming, potentially making it a promising tool for marker-assisted selection of grooming behavior in the future [16,17].

Most of the available data are derived from studies on *A. mellifera*, necessitating further research to elucidate the characteristics associated with auto-grooming behavior in *A. cerana* [18]. Moreover, the molecular mechanisms underlying the grooming behavior of *A*. *cerana* bees remain to be elucidated. This study aims to identify genes related to auto-grooming behavior (light versus intense) through RNA-seq analysis of the brains in both *A. cerana* and *A. mellifera*. Our results revealed that the differentially expressed mRNAs related to grooming behavior in both *A. cerana* and *A. mellifera* were enriched in the neuroactive ligand–receptor interaction pathway, inferring a potential role for neural regulation of grooming behavior.

## 2. Materials and Methods

### 2.1. Honeybee and Mites Collection

The healthy adult worker bees of *A. mellifera* and *A. cerana* were collected from six different colonies at Fujian Agriculture and Forestry University, Fuzhou, China. *Varroa* mites were obtained from four mite-infested bee colonies found in the Fuqing bee farm of Fuzhou, China, and reared separately from healthy bee colonies. Three frames containing capped broods were randomly selected from three different colonies. Twenty-four hours before the bee’s emergence, the frames were individually placed in a screened cage and stored in an incubator at 34 ± 0.5 °C with 75 ± 2% relative humidity (RH). Newly emerged bees were individually marked on the thorax using a marker pen with distinct colors (bees of the same age were assigned the same color) and introduced into their original colony for natural rearing. At intervals of 5, 7, 9, 11, 13, and 15 days post-emergence, marked bees were selected from the colony and transferred into a honeybee feeding box containing 50% sugar water, allowing the bees to feed freely. Approximately one hundred color-marked bees per age group were collected and transported to the laboratory for behavioral assays.

*Varroa* mites were collected using the icing sugar method. Mites were shaken onto a damp paper towel and rinsed with a drop of distilled water. Living mites were transferred to Petri dishes with moist pieces of paper towel and placed in a laboratory room kept at 30 ± 0.5 °C. These mites were promptly utilized for artificially infesting the color-marked bees, ensuring their immediate attachment to the host, as only active vital mites were selected for the bioassays.

### 2.2. Auto-Grooming Behavior Experiment

Auto-grooming assay methods were performed as previously described by Guzman-Novoa [15], with minor modifications. Each worker bee was placed inside a Petri dish (90 mm × 15 mm) covered with a perforated lid and left there for 2 min to acclimatize to the environment. Furthermore, a mite was gently transferred onto the bee’s thorax using a small paintbrush. To evaluate the grooming intensity of bees, each bee was observed for 3 min to record the time of the first reaction to the mite. We recorded the extent of grooming by the bees’ legs to remove mites. An attempt to groom was defined as an uninterrupted period during which grooming behavior was observed, ending when the bees paused. The data also included whether the grooming successfully removed the mite. Bees that successfully removed the mite within 3 min were considered strong groomers. In addition, the worker bees unable to groom the mite were categorized based on their behavioral observations and statistical data. Those that slowly moved without utilizing more than two legs for removal were classified as weak groomers, while those exhibiting shaking or wiping motions and employing three or more legs for rapid removal were labeled as strong groomers. After 3 min of trials, each bee was flash-frozen using liquid nitrogen and stored at −80 °C for further gene expression analysis.

### 2.3. RNA Extraction, Library Construction and Data Quality Control

The collected honeybee brains were dissected on the ice with a microscope to remove the hypopharyngeal glands, salivary glands, three simple eyes, and two compound eyes. Total RNA was extracted from the brains of the samples (each pooled from 10 honeybees) using a Trizol reagent kit (Invitrogen, Carlsbad, CA, USA) according to the manufacturer’s protocol. The extracted total RNA samples were assessed as follows: (1) 1% agarose gel electrophoresis was used to analyze the RNA integrity of the samples and whether there was DNA contamination; (2) NanoDrop was used to detect the RNA purity (OD260/280); (3) Qubit 2.0 was used to quantify the RNA concentration accurately; and (4) Agilent 2100 (Agilent, Santa Clara, CA, USA) was used to assess RNA integrity for library construction purposes. After total RNA extracted, eukaryotic mRNA was enriched using Oligo (dT) Beads. Then, the enriched mRNA was fragmented into fragments using fragmentation buffer and reverse transcribed into cDNA using NEBNext Ultra RNA Library Prep Kit for Illumina (NEB #7530, New England Biolabs, Ipswich, MA, USA). The purified double-stranded cDNA fragments were end-repaired, and a base was added and ligated to Illumina sequencing adapters. The ligation reaction was purified with AMPure XP beads (1.0×). Ligated fragments were subjected to size selection via agarose gel electrophoresis and amplified polymerase chain reaction (PCR). The resulting cDNA library was sequenced using Illumina Hiseq^TM^ 4000 by Gene Denovo Biotechnology Co. (Guangzhou, China). All reads produced in this research have been deposited in the National Centre for Biotechnology Information (NCBI) and can be accessed in the Short Read Archive (SRA) Database under accession PRJNA1100954 (*A. cerana*) and PRJNA1100956 (*A. mellifera*).

### 2.4. Differential Expression Genes Analysis

Based on read count, the reference genome was compared via HISAT2 software, the transcript was reconstructed with Stringtie, and the expression value (FPKM) of all genes in each sample was calculated using RSEM. The FPKM value was used as the gene expression index, and the differential expression was analyzed with DESeq2 software [19]. Differentially expressed genes (DEGs) were filtered based on the threshold of significant differential expression, which was *p* < 0.05, |log2fc| > log2(2). ClusterProfiler R package software, combined with the GO enrichment database and KEGG database, was used for GO enrichment analysis and pathway enrichment analysis of DEGs, with a threshold of *p* < 0.05 being used as the standard of significant enrichment [20].

### 2.5. Homologous Gene Analysis

Homologous genes are genes inherited from a shared ancestor in different species. The corresponding protein sequences were found by translating all the gene sequences of the *A. cerana* and *A. mellifera* genomes; then, blast alignment was performed using the NCBI BLAST-2.6.0 + program. The parameters set by the software were filtered as default parameters. Finally, the homologous gene tables of the two bees were obtained. Combined with the genes *HTR2A* and *SLC17A8* screened by the transcriptome of *A. cerana*, the corresponding protein-coding sequences were searched on NCBI to match the homologous gene table to find the homologous genes in *A. mellifera*.

### 2.6. Real-Time Fluorescence Quantitative PCR Verification

Differentially expressed mRNA genes were randomly selected for qPCR validation, and the housekeeping gene *β-actin* was used as the reference gene. The ten pairs of primers used to amplify the genes evaluated are shown in Table S1. Reverse transcription of cDNA was used as a template for fluorescence quantitative PCR according to the Hieff^®^ qPCR SYBR^®^ Green Master Mix kit instructions. The mixture included Hieff^®^ qPCR SYBR^®^ Green Master Mix reagent: 5 μL; cDNA template: 0.2 μL; primer: 0.4 μL; and ddH_2_O: 4.4 μL.

### 2.7. Statistical Analyses

The rank sum test was used for statistical analysis, and the data were presented as a percentage with a *p*-value < 0.05. Statistical analyses of the qPCR data were performed using SPSS software (IBM) and GraphPad Prism8.0 software. The data were presented as mean ± SD.

## 3. Results

### 3.1. Different Days Analysis of Auto-Grooming Behavior

In the *A. cerana* group, the strong grooming rates of 5-, 7-, 9-, 11-, 13-, and 15-day-old bees were observed to be 16.67%, 33.33%, 71.67%, 51.67%, 46.67%, and 41.67%, respectively. There was a very significant difference in the degree of strong and weak grooming behavior among the six days (*p* < 0.0001), which indicated that the strong and weak grooming behavior of *A. cerana* was closely related to the age of the bees. Comparing 5-day-old bees with those of other ages, there was a significant difference between 5-day-old bees and 9-day-old bees (*p* < 0.0001), between 5-day-old bees and 11-day-old bees (*p* < 0.01), and between 5-day-old bees and 13-day-old bees (*p* < 0.05).

**Figure 1:**
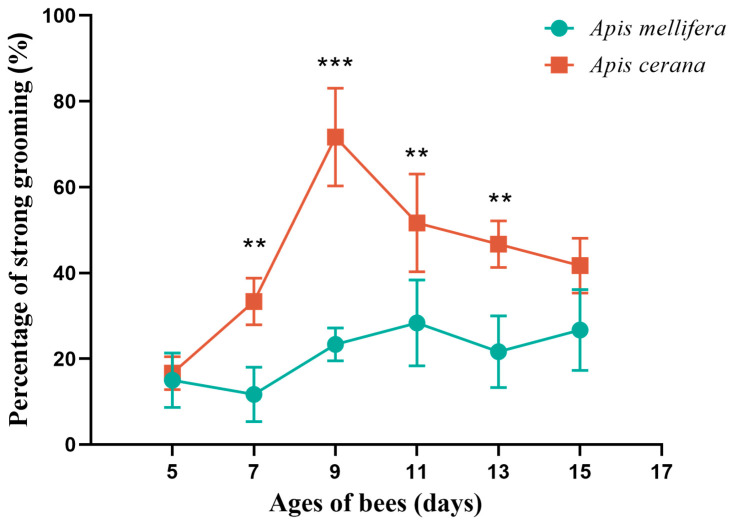
Percentage of strong grooming of *Apis cerana* (*A*. *cerana*) and *Apis mellifera* (*A*. *mellifera*) workers on different days. ** indicates *p* < 0.01, *** indicates *p* < 0.001.

In the *A. mellifera* group, the strong grooming rates of 5-, 7-, 9-, 11-, 13-, and 15-day-old bees were observed to be 15%, 11.67%, 23.33%, 28.33%, 21.37%, and 26.67%, respectively. There was no significant difference in the degree of strong and weak grooming behavior among the 6-day-old bees (*p* > 0.05), indicating that there was no statistical difference between the ages for *A. mellifera*, and no pairwise comparison was performed (Figure 1, Table S2).

The strong and weak grooming behaviors of the two bee species with the same age were compared. At 7, 9, 11 and 13 days old, there were significant differences in the degree of strong and weak grooming behavior between *A. cerana* and *A. mellifera* (*p* < 0.01) (Table S2). This shows that the species of bees may affect the strong and weak grooming behavior. Notably, 9-day-old *A. cerana* showed the greatest difference in grooming behavior compared with other *A. cerana* and *A. mellifera* of the same age. The difference between age and grooming behavior prompted us to select the brains of 9-day-old workers with strong and weak grooming behaviors for further RNA-seq sequencing.

### 3.2. Overview of RNA-Seq Data

A total of 250,423,076 raw reads were obtained from six samples of *A. cerana* via high-throughput sequencing, with the largest number of raw reads in the CW_3 group being 46,726,902. After quality control, a total of 249,364,392 clean reads were produced, with the largest number of CW_3 group being 46,537,632. The proportion of clean reads in the original data of these six databases was more than 99.55%, meeting necessary testing standards. Based on base quality analysis, the Q20 content of the six samples was more than 97% of the total base number, and the Q30 content of all samples was also more than 93%. The GC content of each sample accounted for more than 40% of the total base number. Mapping the clean reads with the genome of *A. cerana* with HISAT software showed a total mapped rate of 84% for the six samples, with the CS_2 group having the highest rate (89.63%). The unique map rate ranged from 80.43% to 86.46%, while multiple mappings ranged from 3.09% to 3.89%. The biological reproducibility of RNA-seq in samples of each group, evaluated via the Pearson correlation evaluation, can be seen in Figure S1, with a correlation above 0.977 for all groups, indicating good repeatability.

In addition, a total of 247,889,298 raw reads were obtained from six samples of *A. mellifera*, with 46,779,512 obtained in the MS_2 group. After quality control, 246,818,962 clean reads were obtained, with 46,580,218 obtained in the MS_2 group. The proportion of clean reads in the original data of the six libraries exceeded 99.55%, meeting necessary detection standards. Based on base quality analysis, the Q20 and Q30 content in all six samples were over 97% and 93%, respectively. The GC content of each sample accounted for more than 39% of the total base number. Mapping the clean reads with the genome of *A. mellifera* with HISAT software showed a total mapping rate of over 71% for all the six samples, with the MS_3 group having the highest rate (81.27%). The unique mapping ranged from 67.89% to 75.93%, and multiple mappings ranged from 3.81% to 5.45%. Figure S2 shows the biological repetitive correlation of each group of samples, with a correlation of more than 0.89, indicating good repeatability. Based on these data, it is concluded that the sequencing results are of good quality and can be used for further analysis.

### 3.3. DEGs Analysis

**Figure 2:**
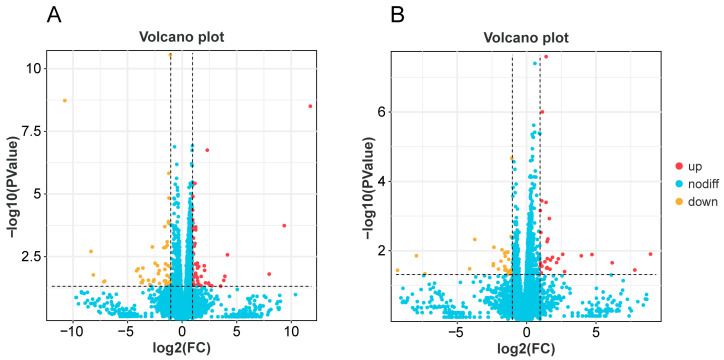
Volcano plots of DEGs in *A. cerana* (**A**) and *A. mellifera* (**B**).

**Figure 3:**
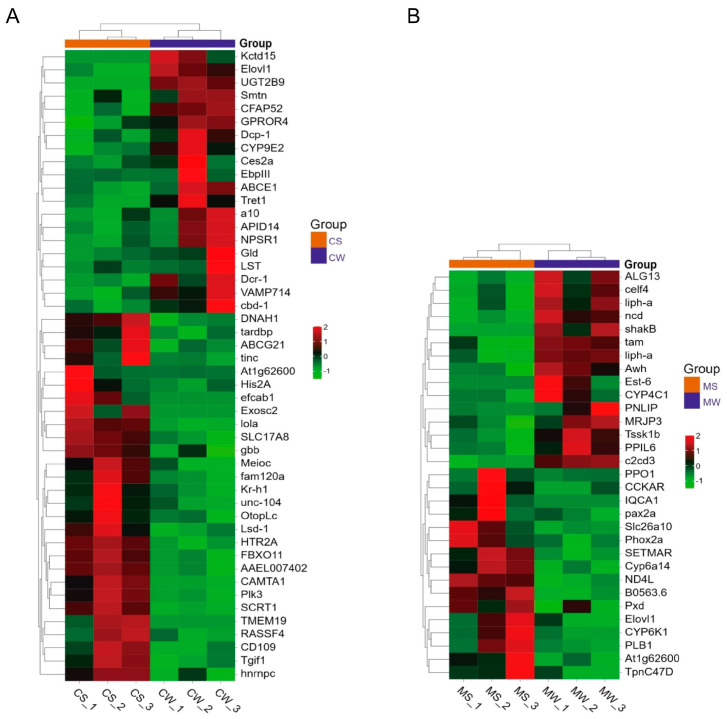
Hierarchical clustering analysis of DEGs in *A. cerana* (**A**) and *A. mellifera* (**B**). CW: weak grooming of *A. cerana*; CS: strong grooming of *A. cerana*; MW: weak grooming of *A. mellifera*; MS: strong grooming of *A. mellifera*.

In *A. cerana*, a total of 9106 genes were identified in CS and CW, of which 8789 genes were common, 8789 genes were group-specific expressions of CS, and 91 genes were detected in CW. A total of 100 differentially expressed genes were found via gene expression analysis, with 51 genes up-regulated and 49 down-regulated in the CS group (Figure 2A). The heat map showed that among these differentially expressed genes, the neurotransmission-related genes *HTR2A* and *SLC17A8*, the calmodulin transcription factor gene *CAMTA1,* and the taste receptor-related gene *OtopLc* were significantly up-regulated in the CS group, with the expression levels more than two times higher than those in the CW group (Figure 3A).

In *A. mellifera*, 7885 genes were identified in MS and MW, 7510 genes were shared, of which 64 genes were specifically expressed in MS and 311 genes were specifically expressed in MW (Figure 2B). There were 63 differentially expressed genes, with 31 were up-regulated and 32 down-regulated. The heat map showed that among these differentially expressed genes, *CCKAR*, *Tpnc47D*, *Ag1t62600,* and other neurotransmitter-related genes, as well as flavin monooxygenase family-related genes, were significantly up-regulated in the MS group, with expression levels of more than two times those in the MW group (Figure 3B).

### 3.4. GO Functional Enrichment and KEGG Pathway Enrichment Analyses of A. cerana and A. mellifera

**Figure 4:**
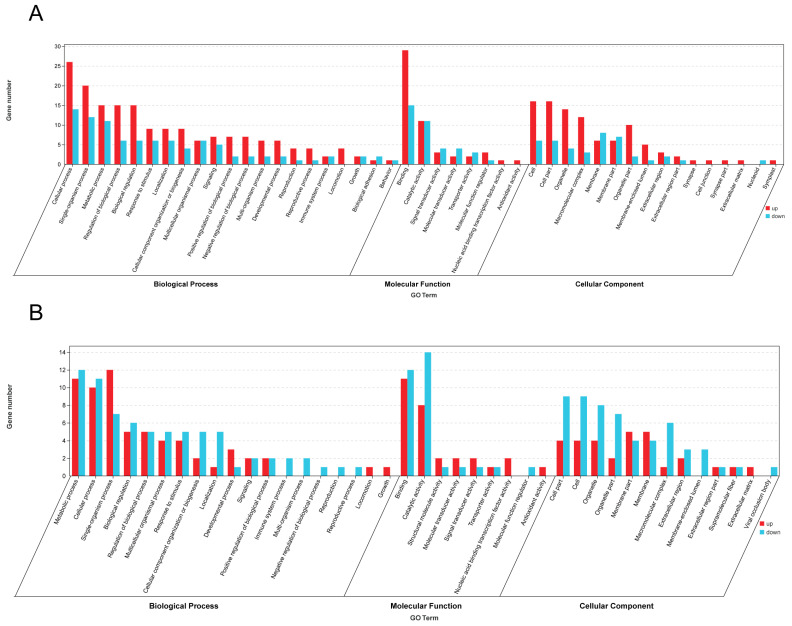
GO classification of DEGs in *A. cerana* (**A**) and *A. mellifera* (**B**).

Differentially expressed genes were systematically functionally annotated based on the significance threshold of *p*-value < 0.05 for significant enrichment. A total of 161 significant enrichment items of the biological process class, 25 significant enrichment items of the molecular function class, and 16 significant enrichment items of the cell components class were identified. The top 20 significant gene ontology (GO) entries are illustrated in Figure 4A below. The number of differentially expressed genes in *A. cerana* enriched in the biological processes group was the highest (268), among which, 26 up-regulated genes were most enriched in the cellular processes group. The down-regulated genes were mainly concentrated in the cellular process group, with a total of 14 genes. A total of 90 genes were enriched in the molecular function group, with 29 up-regulated genes enriched in binding and 15 down-regulated genes enriched in the binding category. There were 136 genes enriched in cell components, with 16 up-regulated genes mainly enriched in the cell and 8 down-regulated genes most enriched in the cell. The GO functional annotation analysis revealed that *SLC17A8* was predominantly enriched in the single-organism cellular process, whereas *HTR2A* was particularly enriched in the intrinsic component of membrane and transmembrane signaling receptor activity. Additionally, the cell surface receptor signaling pathway was identified as the main enriched pathway of *HTR2A*.

**Figure 5:**
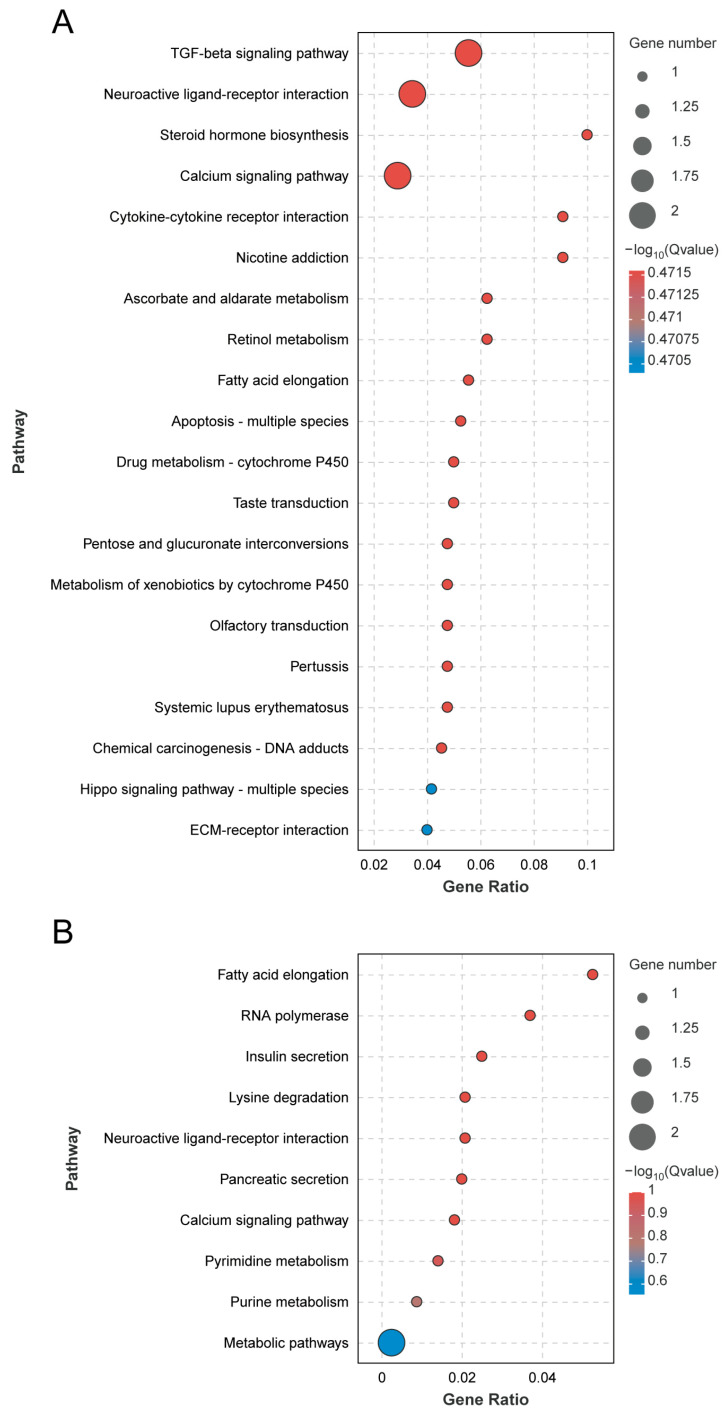
KEGG significantly enriched pathways in *A. cerana* (**A**) and *A. mellifera* (**B**).

According to the KEGG pathway analysis of differentially expressed genes, 57 differentially expressed genes were annotated to 53 pathways. Among them, the most enriched genes were of the signal transduction secondary classification, which was enriched to eight genes. The first 20 pathways of the greatest differential enrichment are listed in Figure 5A. The TGF-β signaling pathway and the neuroactive ligand–receptor interaction pathway are significantly enriched. Focusing on the analysis of the neuroactive ligand–receptor interaction pathway and the calcium signaling pathway, these two pathways are enriched in the *HTR2A* gene, which is up-regulated in the CS.

The concentration of differentially expressed genes in the *A. mellifera* group showed that 136 genes were enriched in biological processes, and 12 up-regulated genes were enriched in the single-organism process. The down-regulated genes were mainly concentrated in the metabolic process group, with 12 in total. A total of 60 genes were enriched in the molecular function group, among which, 11 genes were enriched in binding, and 14 genes were enriched in catalytic activity. There were 86 genes enriched in the cell component, with five up-regulated genes mainly enriched in the membrane part, and nine down-regulated genes most enriched in cells. Altogether, 54 significant enrichment items of the biological process class, 12 significant enrichment items of the molecular function class, and 19 significant enrichment items of the cell group class were screened. The top 20 GO entries of significance are shown in Figure 4B. Through GO functional annotation analysis, *CCKAR* was mainly enriched into the intrinsic component of the membrane, G-protein-coupled peptide receptor activity, and cell surface receptor signaling pathway. *TpnC47D* is mainly enriched in calcium ion binding and protein binding.

According to the KEGG pathway analysis of differentially expressed genes, 20 pathways were annotated to differentially expressed genes. Among them, the most enriched genes are the global overview maps of secondary classification, which were enriched in two genes. The top 20 pathways of the most differential enrichment are listed in Figure 5B, with fatty acid elongation and RNA polymerase being the most significant. Focusing on the analysis of neuroactive ligand–receptor interaction and the calcium signaling pathway, these two pathways are enriched in the *CCKAR* gene, which is up-regulated in the WS.

### 3.5. qPCR Analysis

**Figure 6:**
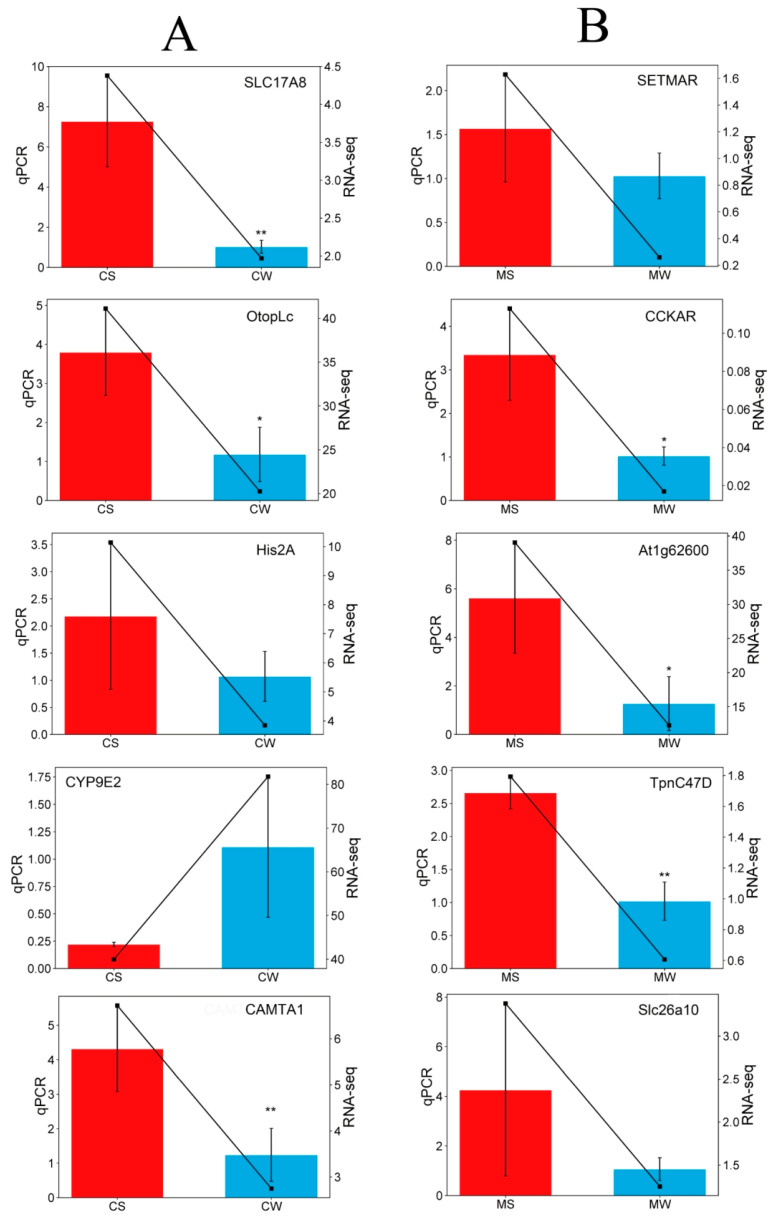
Comparison of RNA-Seq and RT-qPCR validation from the strong and weak grooming groups of *A. cerana* (**A**) and *A. mellifera* (**B**).* *p* < 0.05; ** *p* < 0.01.

To verify the accuracy of RNA-seq results, five differentially expressed genes were selected from the *A. cerana* transcriptome and the *A. mellifera* transcriptome, respectively, for real-time fluorescence quantitative PCR validation. As shown in Figure 6, among the five differential genes selected in the *A. cerana* group, the expression level of *CYP9E2* in bees in the strong grooming group was lower than that in the weak grooming group, while the expression of *CAMTA1*, *Otoplc*, *His2A,* and *SLC17A8* was up-regulated in the strong grooming group. Among the five differential genes selected in the *A. mellifera* group, *At1g62600*, *CCKAR*, *SLC26A10*, *SETMAR,* and *Tpnc47D* were up-regulated in the strong grooming group, which was higher than that in the weak grooming group. The results of fluorescence quantitative PCR were consistent with the sequencing results, which reflected the reliability of RNA-seq.

### 3.6. Homologous Gene Analysis

*HTR2A* and *SLC17A8* genes were screened via RNA-seq analysis of *A. cerana*, and the corresponding protein-coding sequences *XM_028664372.1* and *XM_017052091.2* were queried on the NCBI. According to the homologous gene table of the two bee species, the corresponding homologous genes of *A. mellifera* were found as follows (Table S4): two homologous genes correspond to *HTR2A* in *A. cerana*; and one homologous gene corresponds to *SLC17A8* in *A. mellifera*. Finally, the homologous gene protein sequences with the highest score, *XM_026443084.1* and *XM_016914051.2*, were found according to the identity and bit score of sequence alignment, and the western bee genes *HTR2A* and *SLC17A7* were found on NCBI. Analysis of the trend of expression of these two genes revealed that the expression of *HTR2A* and *SLC17A7* was up-regulated in *A. mellifera*, consistent with the RNA-seq results of *A. cerana*.

## 4. Discussion

This study highlights a significant relationship between the strength of grooming behavior in *A. cerana* and the age of the bees (*p* < 0.0001). While no notable difference was observed in the grooming behavior of *A. mellifera* at 6 days old (*p* > 0.05), the rate of strong grooming exhibited a degree of variation over time. This phenomenon may be attributed to the shifting roles undertaken by worker bees as they progress through different stages within the colony. For instance, worker bees aged 4 to 6 days old are primarily engaged in feeding large larvae and adjusting pollen. Additionally, besides feeding the queen and the larvae at 1 to 3 days old, workers aged 6 to 12 days old are also responsible for cleaning the nest and removing diseased larvae and bees. From 13 to 18 days of age, they gradually transition from nurse bees to house bees and take on responsibilities such as beeswax secretion and nesting. At 9 days old, compared with the 5-, 7-, and 11-day nursing bees, 11-day workers begin to change to house bees, and the work of nursing bees gradually decreases. Workers at 5 and 7 days of age are in the early learning state of nursing bees, while workers at 9 days old have become adept at cleaning nests and removing diseased larvae and individuals. Thus, at day 9, there was a high proportion of strong grooming. Panasiuk et al. demonstrated that hygienic behaviors involve bees across various age ranges but are more commonly observed between 6 to 10 and 16 to 21 of age [21]. Pettis and Pankiw studied the role of grooming behavior in bees based on the bees’ age, revealing that approximately 90% of grooming behavior occurred in bees between 5 and 15 days old [22]. Nedjma conducted an individual-level study on the grooming behavior of resistant versus susceptible bees at 4, 7, 15, and 21 days of age at the individual level. Their findings showed that the resistant 7-day-old bees exhibited the highest mite removal rate, while those at the age of 21 days displayed the lowest mite removal rate due to the division of labor within the bee colony [23].

The neuroactive ligand–receptor interaction pathway covers a variety of ligands and receptors on the plasma membrane and participates in the regulation of signal transduction inside and outside the cell. In this pathway, it was found that the differentially expressed gene *HTR2A*, which belongs to the biogenic amine subclass receptor in the neuroactive ligand receptor interaction pathway, was up-regulated. Serotonin (5-HT), a biogenic amine acting as a messenger in most animals, plays an essential role in regulating various physiological, cognitive, and behavioral functions [24,25]. Studies have shown that serotonin regulates the secretory process [26], development [27], circadian rhythm [28], learning, and memory in insects [29,30]. Serotonin is also abundant in the central nervous system of bees [31,32]. In adult bees specifically, serotonin content increases with age, while foraging bees exhibit higher levels compared to nurse bees. The serotonin content also differs in age-matched bees with different divisions of labor [33]. However, limited research has been conducted regarding the specific roles of 5-HT receptor subtypes in bees. It has been reported that the *5-HT1A* receptor is highly expressed in the brain regions of honeybees known to be involved in visual information processing, and it is confirmed that serotonin is involved in the regulation of phototaxis behavior in honeybees, and the *5-HT1A* receptor may be the medium of this regulation [34]. In another study, Perrot-Minnot et al. injected the crustacean amphipod *Gammarus pulex* with both 5-HT2A receptor antagonists and agonists to investigate the organism’s phototactic response. They discovered that serotonin-induced phototactic behavior could be inhibited by the antagonists. These findings indicate that the positive phototaxis triggered by serotonin might be facilitated through the activation of serotonin receptor type 2. The *5-HT2A* affects neural activity, perception, cognition, and emotion and plays a role in regulating behavior [35]. In summary, 5-HT receptors affect the behavior of insects. The differential gene *HTR2A* was up-regulated in the brains of bees in the strong grooming group, indicating that the anti-mite grooming behavior of *A. cerana* may also be related to 5-HT receptors.

The synapse is a place for communication between neurons and is the basis of the neural circuitry that controls the cognition and behavior of all animals. Chemical synapses are specialized asymmetric connections between presynaptic neurons and postsynaptic targets that are formed through a series of distinct cellular and subcellular events under the control of complex signaling networks. Once established, synapses promote neurotransmission by mediating the organization and the fusion of synaptic vesicles [36]. Glutamate plays a key role in the central nervous system as a major excitatory neurotransmitter that regulates the majority of excitatory transmission between neurons. This regulation affects various functions of the brain, such as cognition, behavior, memory, and learning [37]. *Drosophila* neuromuscular junction (NMJ) is an asymmetric chemical synapse formed between *Drosophila* motor neurons and muscle cells that are similar to glutamatergic synapses in many functions. It is widely used to study the regulation of glutamate receptors [38]. In the KEGG pathway analysis of *A. cerana* RNA-seq date, the differentially expressed gene *SLC17A8* was enriched in the glutamatergic synaptic pathway and up-regulated in the pathway. Based on analogy with the *Drosophila* neuromuscular junction between motor neurons and muscle cells, we speculate that the up-regulation of *SLC17A8* may regulate the intense grooming behavior of bees; thus *SLC17A8*, was screened as a potential anti-mite grooming behavior gene of *A. cerana*.

KEGG pathway analysis of differentially expressed genes in *A. mellifera* revealed that the cholecystokinin receptor *CCKAR* was enriched in the neuroactive ligand–receptor interaction pathway. When an animal is in different behavioral states and faces more than one stimulus, multiple decision-making processes are involved in making the right behavioral choices [39]. For example, CCK inhibits gastric emptying and food intake by stimulating the secretion of pancreatic enzymes and the contraction of the gallbladder [40]. In addition, CCK is a potent neurotransmitter in both the central and peripheral nervous systems, and CCK released by brain neuroendocrine cells has regulatory functions in nociception, memory and learning processes, panic, and anxiety [41]. By comparing the behavior of transgenic mice overexpressing progastrin with that of normal mice, previous studies have shown that the transgenic mice exhibited significantly increased aggression, motor activity, and anxiety-related behaviors. Further analysis revealed that the expression of the CCK2R receptor and the 5-HT1A receptor in the hypothalamus of the mice was up-regulated, indicating that the behavioral changes in the mice were influenced by the up-regulation of these genes [42]. In insects, there is also a linearly homologous neuropeptide DSK with a similar function to CCK, which acts as a satiety signal in flies, crickets, locusts, cockroaches, and other insects [43]. Some researchers have confirmed that DSK activates the *CCKLR-17D1* receptor and promotes movement and escape behavior in *Drosophila* larvae [44]. The above studies suggest that cholecystokinin and its receptors affect motor behavior in insects. The strong grooming behavior of bees under the stress of *Varroa* mites can be considered an anxiety attack behavior similar to that exhibited in transgenic mice. It is speculated that the gene *CCKAR* may regulate the *Varroa*-resistant grooming behavior of *A. mellifera*.

The strong grooming behavior of bees is a rapid multi-part movement, which requires the sliding of multiple feet, the vibration of wings, and the twisting of the body. And these movements are bound to produce muscle contractions. Muscle contraction is produced by the relative sliding of actin and the tropomyosin–troponin complex [45]. Troponin consists of three components, each with a specific function: troponin I inhibits the ATPase activity of actin; troponin T provides the binding of troponin to tropomyosin [46]; and troponin C is responsible for the binding of Ca^2+^. When the intracellular Ca^2+^ concentration is increased, calcium signaling is promoted, thereby enhancing the interaction between actin and myosin [47,48]. In the comparison of strong and weak grooming behavior of *A. mellifera*, the actin C gene *TpnC47D* was significantly up-regulated in the strong grooming group, and KEGG analysis showed that this gene was significantly enriched in the calcium ion pathway. It can be speculated that when *A. mellifera* carries out rapid and intense muscle activity, the concentration of Ca^2+^ in honeybee cells increases, and *TpnC47D* is also up-regulated to promote the interaction between actin and myosin, forming muscle contraction and promoting the grooming behavior of honeybees.

## 5. Conclusions

This study observed the auto-grooming behavior and analyzed the RNA sequence of brains from two closely related honeybee species infested with mites. Compared to *A. mellifera*, *A. cerana* exhibited a higher frequency of strong auto-grooming behavior, which may explain its stronger resistance to mites. In conclusion, this study showed that the DEGs in *A. mellifera* and *A. cerana* were enriched in the neuroactive ligand–receptor interaction pathway. By analyzing and screening the transcriptome data of *A. cerana*, we identified the genes *HTR2A* and *SLC17A8*, which are related to anti-mite grooming behavior, and we analyzed their homology, obtaining the corresponding *A. mellifera* homologous genes *HTR2A* and *SLC17A7*. Combined with differential gene expression, functional annotation, and homologous gene analysis, it is speculated that *CCKAR*, *TpnC47D*, *HTR2A*, and *SLC17A7* are the anti-mite grooming-behavior-related genes. Moreover, *CCKAR* and *HTR2A* regulate many behaviors in other insects. These results indicated that the instance of grooming behavior in honeybees affected the gene expression profile and expression pattern in honeybee brains. Further research is required to clarify the mechanisms underlying these changes and elicit their functions. At the same time, RNAi and other molecular biology techniques can be employed to verify and explore the genes identified in this screening study and to explore the molecular mechanisms underlying *Varroa*-resistant grooming behavior in honeybees.

## Data Availability

Data are contained within the article or Supplementary Material. The original contributions presented in the study are included in the article/Supplementary Material. Further inquiries can be directed to the corresponding authors.

## Supplementary Materials

The following supporting information can be downloaded at https://www.mdpi.com/article/10.3390/genes15060763/s1: Figure S1: Pearson correlation analysis between samples of *A. cerana*; Figure S2: Pearson correlation analysis between samples of *A. mellifera*; Table S1: Primer sequences of 11 genes used in qPCR; Table S2: Different ages analysis of auto-grooming behavior; Table S3: RNA-seq data summary and mapped information of *A. cerana* and *A. mellifera*; Table S4: *A. cerana* and *A. mellifera* gene *HTR2A* and gene *SLC17A8* homologous.

## References

[1] Traynor K.S., Mondet F., de Miranda J.R., Techer M., Kowallik V., Oddie M.A.Y., Chantawannakul P., McAfee A. (2020). *Varroa Destructor*: A Complex Parasite, Crippling Honey Bees Worldwide. Trends Parasitol..

[2] Warner S., Pokhrel L.R., Akula S.M., Ubah C.S., Richards S.L., Jensen H., Kearney G.D. (2024). A scoping review on the effects of *Varroa* Mite (*Varroa Destructor*) on global honey bee decline. Sci. Total Environ..

[3] Techer M.A., Roberts J.M.K., Cartwright R.A., Mikheyev A.S. (2022). The first steps toward a global pandemic: Reconstructing the demographic history of parasite host switches in its native range. Mol. Ecol..

[4] Rosenkranz P., Aumeier P., Ziegelmann B. (2010). Biology and control of *Varroa Destructor*. J. Invertebr. Pathol..

[5] Vilarem C., Piou V., Vogelweith F., Vétillard A. (2021). *Varroa Destructor* from the Laboratory to the Field: Control, Biocontrol and IPM Perspectives—A Review. Insects.

[6] Morfin N., Goodwin P.H., Guzman-Novoa E. (2023). *Varroa Destructor* and its impacts on honey bee biology. Front. Bee Sci..

[7] Le Conte Y., Meixner M., Brandt A., Carreck N., Costa C., Mondet F., Ralph B. (2020). Geographical distribution and selection of european honey bees resistant to *Varroa Destructor*. Insects.

[8] Luis A.R., Grindrod I., Webb G., Piñeiro A.P., Martin S.J. (2022). Recapping and mite removal behaviour in Cuba: Home to the world’s largest population of *Varroa*-resistant European honeybees. Sci. Rep..

[9] Grindrod I., Martin S.J. (2023). *Varroa* resistance in *Apis Cerana*: A review. Apidologie.

[10] Guichard M., Dietemann V., Neuditschko M., Dainat B. (2020). Advances and perspectives in selecting resistance traits against the parasitic mite *Varroa Destructor* in honey bees. Genet. Sel. Evol..

[11] Invernizzi C., Zefferino I., Santos E., Sánchez L., Mendoza Y. (2015). Multilevel assessment of grooming behavior against *Varroa Destructor* in Italian and Africanized honey bees. J. Apic. Res..

[12] van Alphen J.J.M., Fernhout B.J. (2020). Natural selection, selective breeding, and the evolution of resistance of honeybees (*Apis Mellifera*) against Varroa. Zool. Lett..

[13] Pritchard D.J. (2016). Grooming by honey bees as a component of varroa resistant behavior. J. Apic. Res..

[14] Nganso B.T., Fombong A.T., Yusuf A.A., Pirk C.W.W., Stuhl C., Torto B. (2017). Hygienic and grooming behaviors in African and European honeybees-New damage categories in *Varroa Destructor*. PLoS ONE.

[15] Guzman-Novoa E., Emsen B., Unger P., Espinosa-Montaño L.G., Petukhova T. (2012). Genotypic variability and relationships between mite infestation levels, mite damage, grooming intensity, and removal of *Varroa Destructor* mites in selected strains of worker honey bees (*Apis Mellifera* L.). J. Invertebr. Pathol..

[16] Arechavaleta-Velasco M.E., Alcala-Escamilla K., Robles-Rios C., Tsuruda J.M., Hunt G.J. (2012). Fine-scale linkage mapping reveals a small set of candidate genes influencing honey bee grooming behavior in response to *Varroa* Mites. PLoS ONE.

[17] Hamiduzzaman M.M., Emsen B., Hunt G.J., Subramanyam S., Williams C.E., Tsuruda J.M., Guzman-Novoa E. (2017). Differential Gene Expression Associated with Honey Bee Grooming Behavior in Response to *Varroa* Mites. Behav. Genet..

[18] Peng Y.-S., Fang Y., Xu S., Ge L. (1987). The resistance mechanism of the Asian honey bee, *Apis Cerana* Fabr., to an ectoparasitic mite, *Varroa Jacobsoni* Oudemans. J. Invertebr. Pathol..

[19] Love M.I., Huber W., Anders S. (2014). Moderated estimation of fold change and dispersion for RNA-Seq data with DESeq2. Genome Biol..

[20] Robinson M.D., McCarthy D.J., Smyth G.K. (2010). edgeR: A Bioconductor package for differential expression analysis of digital gene expression data. Bioinformatics.

[21] Panasiuk B., Skowronek W., Bieńkowska M., Gerula D., Węgrzynowicz P. (2010). Age of worker bees performing hygienic behaviour in a honeybee colony. J. Apic. Sci..

[22] Pettis J.S., Pankiw T. (1998). Grooming behavior by *Apis Mellifera* L. in the presence of *Acarapis Woodi* (Rennie) (Acari: Tarsonemidae). Apidologie.

[23] Dadoun N., Nait-Mouloud M., Mohammedi A., Sadeddine Zennouche O. (2020). Differences in grooming behavior between susceptible and resistant honey bee colonies after 13 Years of natural selection. Apidologie.

[24] Weiger W.A. (1997). Serotonergic modulation of behaviour: A phylogenetic overview. Biol. Rev. Camb. Philos. Soc..

[25] Dag U., Nwabudike I., Kang D., Gomes M.A., Kim J., Atanas A.A., Bueno E., Estrem C., Pugliese S., Wang Z. (2023). Dissecting the functional organization of the *C. elegans* serotonergic system at Wwhole-brain scale. bioRxiv.

[26] Walz B., Baumann O., Krach C., Baumann A., Blenau W. (2006). The aminergic control of cockroach salivary glands. Arch. Insect Biochem. Physiol..

[27] Colas J.F., Launay J.M., Kellermann O., Rosay P., Maroteaux L. (1995). *Drosophila* 5-HT_2_ serotonin receptor: Coexpression with fushi-tarazu during segmentation. Proc. Natl. Acad. Sci. USA.

[28] Yuan Q., Lin F., Zheng X., Sehgal A. (2005). Serotonin Modulates Circadian Entrainment in *Drosophila*. Neuron.

[29] Sitaraman D., Zars M., LaFerriere H., Chen Y.-C., Sable-Smith A., Kitamoto T., Rottinghaus G.E., Zars T. (2008). Serotonin is necessary for place memory in *Drosophila*. Proc. Natl. Acad. Sci. USA.

[30] Coray R., Quednow B.B. (2022). The role of serotonin in declarative memory: A systematic review of animal and human research. Neurosci. Biobehav. Rev..

[31] Schürmann F.W., Klemm N. (1984). Serotonin-immunoreactive neurons in the brain of the honeybee. J. Comp. Neurol..

[32] Seidel C., Bicker G. (1996). The developmental expression of serotonin-immunoreactivity in the brain of the pupal honeybee. Tissue Cell.

[33] Schulz D.J., Robinson G.E. (1999). Biogenic amines and division of labor in honey bee colonies: Behaviorally related changes in the antennal lobes and age-related changes in the mushroom bodies. J. Comp. Physiol. A.

[34] Thamm M., Balfanz S., Scheiner R., Baumann A., Blenau W. (2010). Characterization of the 5-HT_1A_ receptor of the honeybee (*Apis Mellifera*) and involvement of serotonin in phototactic behavior. Cell Mol. Life Sci..

[35] Perrot-Minnot M.-J., Dion E., Cézilly F. (2013). Modulatory effects of the serotonergic and histaminergic systems on Light in the crustacean *Gammarus Pulex*. Neuropharmacology.

[36] Chou V.T., Johnson S.A., Van Vactor D. (2020). Synapse development and maturation at the *drosophila* neuromuscular junction. Neural Dev..

[37] Davanger S., Manahan-Vaughan D., Mulle C., Storm-Mathisen J., Ottersen O.P. (2009). Protein trafficking, targeting, and interaction at the glutamate synapse. Neuroscience.

[38] Sun M., Xie W. (2012). Cell adhesion molecules in *Drosophila* synapse development and function. Sci. China Life Sci..

[39] Wu S., Guo C., Zhao H., Sun M., Chen J., Han C., Peng Q., Qiao H., Peng P., Liu Y. (2019). Drosulfakinin signaling in *fruitless* circuitry antagonizes P1 neurons to regulate sexual arousal in *Drosophila*. Nat. Commun..

[40] Dockray G.J. (2009). Cholecystokinin and gut-brain signalling. Regul. Pept..

[41] Rehfeld J.F., Friis-Hansen L., Goetze J.P., Hansen T.V.O. (2007). The biology of cholecystokinin and gastrin peptides. Curr. Top. Med. Chem..

[42] Li Q., Deng X., Singh P. (2007). Significant Increase in the aggressive behavior of transgenic mice overexpressing peripheral progastrin peptides: Sssociated changes in CCK_2_ and serotonin receptors in the CNS. Neuropsychopharmacology.

[43] Nässel D.R., Williams M.J. (2014). Cholecystokinin-like peptide (DSK) in *Drosophila*, not only for satiety signaling. Front. Endocrinol..

[44] Chen X., Peterson J., Nachman R.J., Ganetzky B. (2012). Drosulfakinin activates CCKLR-17D1 and promotes larval locomotion and escape response in *Drosophila*. Fly.

[45] Farah C.S., Reinach F.C. (1995). The Troponin complex and regulation of muscle contraction. FASEB J..

[46] Filatov V.L., Katrukha A.G., Bulargina T.V., Gusev N.B. (1999). Troponin: Structure, properties, and mechanism of functioning. Biochemistry.

[47] Lehman W., Bullard B., Hammond K. (1974). Calcium-dependent myosin from insect flight muscles. J. Gen. Physiol..

[48] Vibert P., Craig R., Lehman W. (1997). Steric-model for activation of muscle thin filaments. J. Mol. Biol..

