# Supplementary material for: Comparison of Brain Gene Expression Profiles Associated with Auto-Grooming Behavior between Apis cerana and Apis mellifera Infested by Varroa destructor
Source: Genes (Basel). 2024 Jun 11;15(6):763. doi: 10.3390/genes15060763 (PMC11202474; doi:10.3390/genes15060763)
Supplement: Supplementary file 1 [file genes-15-00763-s001.zip › genes-3010704-supplementary.pdf]

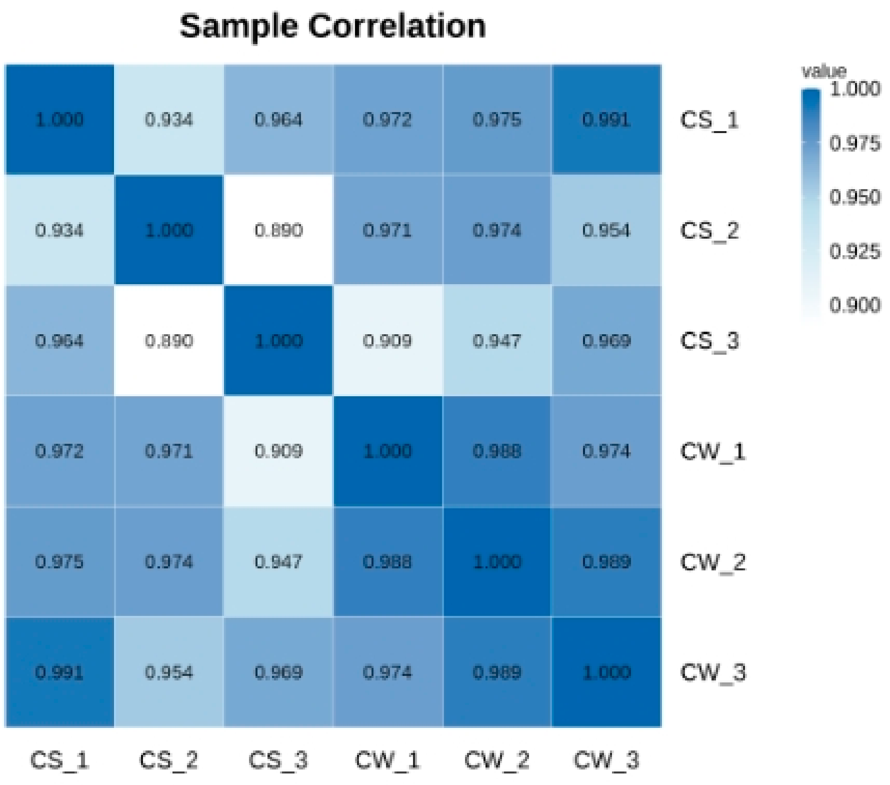

Figure S1. All sample pearson of *A.cerana*.

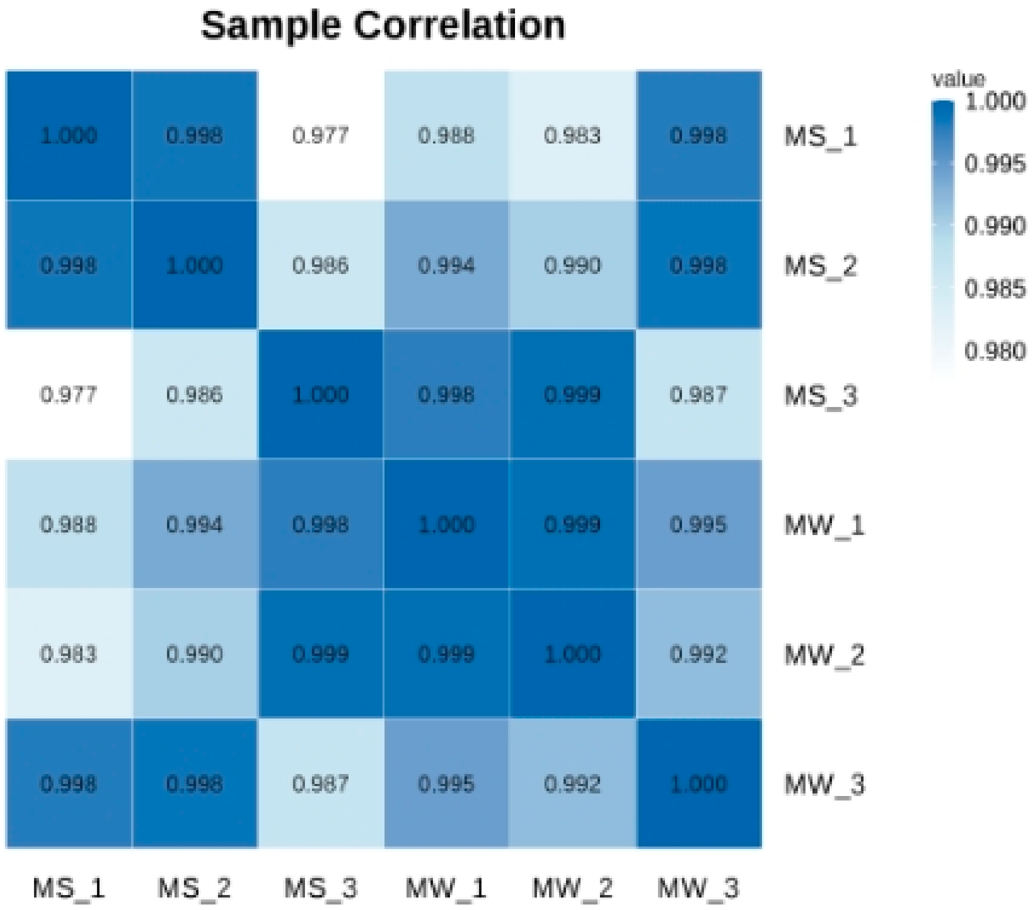

Figure S2. All sample pearson of *A. mellifera*.

Table S1. Primer sequences of 11 genes used in qPCR.

mRNA name	Gene ID	Primer Sequences (5′to 3′)
β-Actin	NM_001185146.1	Forward：CCTAGCACCATCCACCATGAA<br>Reverse：GAAGCAAGAATTGACCCACCAA
CYP9E2	*LOC108004123*	Forward：AAACATGGGCTCGTTGGTC<br>Reverse：GGCGTGGTGAACTCGTAAA
CAMTA1	*LOC107995465*	Forward：ATCTACGGCTGTTATGTGC<br>Reverse：GTTTAGGTAGTGAACGAGGAC
OtopLc	*LOC108002750*	Forward：GAATCTGTCGGTCTGGTTGA<br>Reverse：CGTGGTGGTAGTGGTTGTG
SLC17A8	*LOC107994928*	Forward：GGTATGTGCGGGTTGATTT<br>Reverse：GCCATTTGGGTTTGTCCTT
His2A	*LOC108003508*	Forward：GGCTGCTGCAGTGTTGGAAT<br>Reverse：GCAAGTTGAAGATGACGAGGTA
At1g62600	*LOC726418*	Forward：CGGCGGAACTTGGATATACAC<br>Reverse：CTGTGATGAACGAACGACGAT
SETMAR	*LOC113218652*	Forward：CACACGCATCTTTGGTCACT<br>Reverse：AGGTCAGGACTATATGGTGGAT
TpnC47D	*LOC494501*	Forward：TGCCTGCCACTTCCAGAG<br>Reverse：TCAGCGATCATTCCGTCCAT
SLC26A10	*LOC100576446*	Forward：GGTAGCAACTTACAGCACTCAA<br>Reverse：ACCGCAGTAGCAACTTCCA
CCKAR	*LOC410654*	Forward：CCAACGAGGAGCAACAAGATT<br>Reverse：CCAGCACGATCACGAACAG

Table S2. Different ages analysis of self-grooming behavior.

Groups	*Apis cerana*(n=360)		*Apis mellifera*(n=360)		Z-values	P-values
ages	WG	SG	WG	SG		
5	50（83.33%）	10（16.67%）	51（85%）	9（15%）	-0.249	0.803
7	40（66.67%）	20（33.33%）	53（88.33%）	7（11.67%）	-2.83	0.005**
9	17（28.33%）	43（71.67%）	46（76.67%）	14（23.33%）	-5.279	<0.0001****
11	29（48.33%）	31（51.67%）	43（71.67%）	17（28.33%）	-2.598	0.009**
13	32（53.33%）	28（46.67%）	47（78.33%）	13（21.37%）	-2.875	0.004**
15	35（58.33%）	25（41.67%）	44（73.33%）	16（26.67%）	-1.725	0.085
H-values	41.283		7.718			
p-values	<0.0001****		0.173			

p<0.05*，p<0.01**，p<0.001***.WG: weak grooming; SG: strong grooming.

Table S3. RNA-seq data summary and mapped information of *A. cerana* and *A. mellifera*.

sample	Raw Reads	Clean Reads	GC (%)	Q20 (%)	Q30 (%)	Unique Mapped (%)	Multiple Mapped (%)	Total Mapped (%)	Exon (%)	Intron (%)	Intergenic (%)
CS_1	44334054	44133054	41.26	97.81	93.68	80.43	3.89	84.31	79.06	12.17	8.77
CS_2	37922452	37780584	40.48	98.11	94.41	86.46	3.17	89.63	81.18	12.98	5.85
CS_3	39014690	38845838	40	97.89	93.80	82.89	3.09	85.98	81.86	10.13	8.01
CW_1	43972952	43773054	41.78	98.05	94.21	82.32	3.47	85.79	79.53	13.51	6.96
CW_2	38452026	38294230	41.08	98.17	94.49	83.25	3.81	87.06	81.33	11.81	6.86
CW_3	46726902	46537632	40.34	97.89	93.74	81.72	3.75	85.47	81.45	11.15	7.40
MS_1	40809116	40637580	40.65	98.26	94.65	67.89	3.81	71.70	87.84	10.37	1.79
MS_2	46779512	46580218	40.55	98.02	93.98	72.01	4.26	76.27	86.90	11.03	2.07
MS_3	39892396	39714684	40.18	98.19	94.44	75.93	5.34	81.27	90.85	7.57	1.58
MW_1	36709590	36553108	40.05	98.14	94.36	71.13	3.91	75.04	88.74	8.50	2.76
MW_2	37008178	36848772	39.76	98.26	94.65	74.58	3.66	78.24	88.77	8.79	2.43
MW_3	46690506	46484600	40.17	97.99	93.91	74.80	5.45	80.25	87.96	9.87	2.16

CW: weak grooming of *A. cerana*; CS: strong grooming of *A. cerana*; MW: weak grooming of *A. mellifera*; MS: strong grooming of *A. mellifera*. GC: GC ratio of filtered sequence bases; Q20: the percentage of bases with mass value of more than 20 in the total number of bases in Clean Data; Q30: The percentage of bases with a mass value of more than 30 in the total number of bases in Clean Data..

Table S4. *A. cerana* and *A. mellifera* gene HTR2A and gene SLC17A8 homologous.

Apis_cerana id	*Apis_mellifera* id	identity（%）	alignment length	mismatches	bit score
XM_028664372.1	XM_026443084.1	99.045	733	5	1485
XM_028664372.1	XM_026443085.1	98.609	575	6	1150
XM_017052091.2	XM_016914051.2	99.437	533	3	1108
